# Subthalamic Beta Activity in Parkinson's Disease May Be Linked to Dorsal Striatum Gray Matter Volume and Prefrontal Cortical Thickness: A Pilot Study

**DOI:** 10.3389/fneur.2022.799696

**Published:** 2022-03-23

**Authors:** Florencia Sanmartino, Álvaro J. Cruz-Gómez, Raúl Rashid-López, Elena Lozano-Soto, Fernando López-Sosa, Amaya Zuazo, Jesús Riqué-Dormido, Raúl Espinosa-Rosso, Javier J. González-Rosa

**Affiliations:** ^1^Department of Psychology, University of Cadiz, Cádiz, Spain; ^2^Psychophysiology and Neuroimaging Group, Institute of Biomedical Research Cadiz (INiBICA), Cádiz, Spain; ^3^Department of Neurology, Puerta del Mar University Hospital, Cádiz, Spain; ^4^Department of Radiodiagnostic and Medical Imaging, Puerta del Mar University Hospital, Cádiz, Spain; ^5^Department of Neurosurgery, Puerta del Mar University Hospital, Cádiz, Spain; ^6^Department of Neurology, Jerez de la Frontera University Hospital, Jerez de la Frontera, Spain

**Keywords:** Parkinson's disease, subthalamic nucleus, beta oscillations, local field potentials, cortical thickness, gray matter volume

## Abstract

**Background:**

Excessive oscillations at beta frequencies (13–35 Hz) in the subthalamic nucleus (STN) represent a pathophysiological hallmark of Parkinson's disease (PD), which correlates well with parkinsonian symptoms and is reduced in response to standard disease treatments. However, the association of disease-specific regional gray matter (GM) atrophy or cortical thickness (CT) with the presence of STN beta oscillatory activity has been poorly investigated but is of relevance given the potential of these variables for extracting information about PD pathophysiology. This exploratory study investigated the involvement of regional GM volume and CT in the basal ganglia-cortical network and its potential association with the presence of STN beta oscillatory activity in PD.

**Methods:**

We acquired preoperative GM densities on T1-weighted magnetic resonance imaging scans and we carried out regional estimation of GM volume and CT. LFP activities from the STN were recorded post-operatively in 7 cognitively preserved PD patients off dopaminergic medication undergoing deep-brain stimulation surgery. Oscillatory beta power was determined by power spectral density of 4-min resting state STN LFP activity. Spearman partial correlation*s* and regression analysis were used to screen the presence of STN beta power for their relationship with GM volume and CT measurements.

**Results:**

After controlling for the effects of age, educational level, and disease duration, and after correcting for multiple testing, enhanced STN beta power showed significant and negative correlations between, first, volume of the right putamen and left caudate nucleus, and second, smaller CT in frontal regions involving the left rostral middle frontal gyrus (MFG) and left medial orbitofrontal gyrus. A lower volume in the right putamen and a lower CT in the left MFG demonstrated the strongest associations with increased STN beta power.

**Conclusions:**

These tentative results seem to suggest that STN LFP beta frequencies may be mainly linked to different but ongoing parallel neurodegenerative processes, on the one hand, to GM volume reduction in dorsal striatum, and on the other hand, to CT reduction of prefrontal-“associative” regions. These findings could further delineate the brain structural interactions underpinning the exaggerated STN beta activity commonly observed in PD patients.

## Introduction

A commonly acknowledged way to study oscillatory activity in PD is to record the local field potentials (LFPs), which are thought to represent the summation of local electrical fields near the recording electrodes, through implanted electrodes in the subthalamic nucleus (STN) intraoperatively during or post-operatively after deep-brain stimulation (DBS) surgery (1, 2). The beta rhythm (13–35 Hz) is the longest-studied STN oscillation and has been theorized to play a key role in Parkinson's disease (3). Increased beta oscillations are the most salient LFP event during resting and awake states in PD patients (4), and has been correlated with symptom severity (5). Furthermore, reduction of this excessive beta oscillatory activity by treatment with levodopa (6) and DBS (7) has been linked with motor symptom improvement (8). As a result, there is growing confirmation highlighting the potential utility of STN beta activity as a functional biomarker for PD motor symptom severity and treatment response (9).

There is strong evidence supporting the notion that the progressive degeneration of dopaminergic neurons in the substantia nigra pars compacta (SN) that project to the striatum is a trait of PD pathology (10, 11), although the role of the striatum as a possible source of beta frequency rhythms in PD is still poorly understood (12). In addition, the underlying neural structure(s) and circuit networks accountable for the prominent STN beta oscillations are indeed intensely controversial. Studies have pointed to the dorsal part of the STN as the main source of this pathological synchronization of beta oscillatory activity (13–15), likely influenced by both the interaction of the STN with other basal ganglia structures, particularly the external segment of the globus pallidus (GPe) (16–18), and exogenous or cortical pattering (17, 19, 20).

In this respect, converging studies indicate that the STN is structurally and functionally connected to different cortical and other gray matter (GM) nuclei (21), with significant degeneration and changes in these projection pathways in PD patients (22), which likely impact the structural integrity of their brain networks. Although a variety of studies have reported thinning of both the frontal and posterior cortex (23–25) and increased atrophy in subcortical (particularly caudate and putamen) and cortical networks spanning frontal, temporal and parietal cortices (26–28), disease-specific regional cortical atrophy is still considered to be controversial as a brain marker in PD. In this regard, GM volume reduction or cortical thickness (CT) and their likely association with the presence of beta oscillatory activity have been poorly investigated, despite the attractive relationship between the variables for extracting information regarding cortico-basal ganglia pathophysiology.

In this preliminary study, we explored the presence of STN beta oscillatory activity and the associations with regional GM volume- and surface-based thickness measurements of the basal ganglia–thalamo-cortical regions in PD and proposed a new approach to integrate brain structural changes and STN functional activity that can be used as adjunctive markers to track the effectiveness of disease treatments and disease progression. We speculate that both reduced GM volume (i.e., understood as a hypothetical volume loss or increased atrophy) of basal ganglia nuclei and reduced CT thickness (i.e., understood as a hypothetical increased cortical thinning), particularly from those brain structures influencing STN functioning, may also contribute to abnormal striatal network dynamics and to the mechanism responsible for pathological synchronization of STN oscillations, resulting in enhanced beta frequency oscillations in PD.

## Materials and Methods

### Participants

Nine patients with idiopathic PD, as defined by the Parkinson's UK Brain Bank (29), undergoing bilateral surgical implantation of DBS electrodes in the STN to treat their motor symptomatology were recruited from the Movement Disorders Neurology Outpatient Clinic at the Puerta del Mar University Hospital. Two participants were excluded from the LFP study based on clinical judgment related to the externalization of DBS leads. The remaining seven participants (aged 37–65 years; five males and two females, mean disease duration: 13 years) included in this study exhibited typical motor symptoms, such as bradykinesia, rigidity, or tremor. Each patient's motor condition was evaluated preoperatively and post-operatively by a specialist in movement disorders with the Unified Parkinson's Disease Rating Scale (UPDRS, part III) (30) in the off- and on-medication states. Additionally, none of the patients showed significant cognitive impairment based on Mini-Mental State Examination (MMSE score <24) performance (31) or major depression based on the Beck Depression Inventory (BDI scores <18) (32). Clinical details are summarized in Table 1 (33, 34).

**Table 1:** Demographic and clinical details of the PD patients included in the study.

**ID**	**Age (years)**	**Sex**	**H^**a**^**	***E* (years)**	**Disease duration (years)**	**Predominant symptoms**	**LEDD pre-surgery**	**UPDRS—III**			**MMSE^**c**^**	**BDI II^**c**^**	**Bipolar channels used for analysis**
								**Pre-surgery**		**Post-surgery^**b**^**			
								**On med**	**Off med**	**Off med/off stim**			
1	64	M	R	4	11	Akinetic-rigid	1,075	35	78	49	27	18	R: 1-2, 2-3
2	64	M	R	23	7	Fluctuations, Akinetic-rigid	1,350	38	59	52	29	16	L: 0-1, 1-2, 2-3; R: 0-1, 1-2
3	60	M	R	5	36	Tremor	500	14	67	20	30	18	L: 1-2, 2-3; R: 0-1, 1-2, 2-3
4	65	M	R	11	10	Akinetic-rigid	1,680	20	46	24	29	18	L: 1-2, 2-3; R: 0-1, 1-2, 2-3
5	40	F	R	17	4	Akinetic-rigid, fluctuations	600	14	44	37	28	10	L: 0-1, 1-2, 2-3
6	59	F	R	10	17	Tremor	1,200	13	71	52	30	8	L: 0-1, 1-2, 2-3; R: 0-1
7	37	M	R	10	6	Akinetic-rigid, dystonia	1,560	22	56	35	28	5	L: 0-1, 1-2, 2-3
**Mean**	55.6			11.4	13		1,137.9	22.3	60.1	38.4	28.7	13.3	
***SD***	11.9			6.7	11		451.1	10.3	12.7	13.2	1.1	5.5	

*H, Handedness; E, Education; LEDD, levodopa-equivalent daily dose in mg (33); UPDRS III, Unified Parkinson's Disease Ratings Scale motor part III; on-med, with dopaminergic treatment; off-med, without dopaminergic treatment; off-stim, with STN DBS switched off; MMSE, Mini-Mental State Examination; BDI II, Beck Depression Inventory; F, female; M, male; R, right; L, left; S.D., standard deviation.*

*^a^Handedness was assessed with the Edinburgh Handedness Inventory (34).*

*^b^Post-surgery UPDRS-III score was measured OFF medication and OFF stimulation on the day of LFP recording (2–3 days post-operative).*

*^c^MMSE and BDI-II were evaluated before DBS surgery during ON-med state*.

### Surgical Procedure

Patients were asked to discontinue Parkinson's medications 24 h before DBS surgery. Operative procedures were performed according to the standard approach for bilateral implantation of DBS electrodes into the STN that has been described previously (35). The stereotactic coordinates and trajectories to the simultaneous bilateral STNs were preoperatively targeted using stereotactic magnetic resonance imaging (MRI) on a neuro-navigational platform (StealthStation; Medtronic). All participants received Medtronic 3389 leads (1.27 mm diameter, 1.5 mm contact length, 0.5 mm intercontact spacing) placed in the STNs of both hemispheres. Microelectrode array recordings and intraoperative clinical response to macrostimulation were used to adjust the final DBS electrode placement at the STN.

### LFP Recordings

Following the routine DBS procedure of Puerta del Mar University Hospital, participants underwent an LFP study in the interval (2–3 days) between their DBS electrode implantation surgery and subsequent surgery for the implantation of extension wires to the subcutaneous stimulator. All medication doses of dopaminergic drugs were stopped for at least 12 h in participants, and LFP recordings took place in the clinically defined off state while the participants were comfortably seated in an eyes-open rest condition during ~4 min.

**Figure 1:**
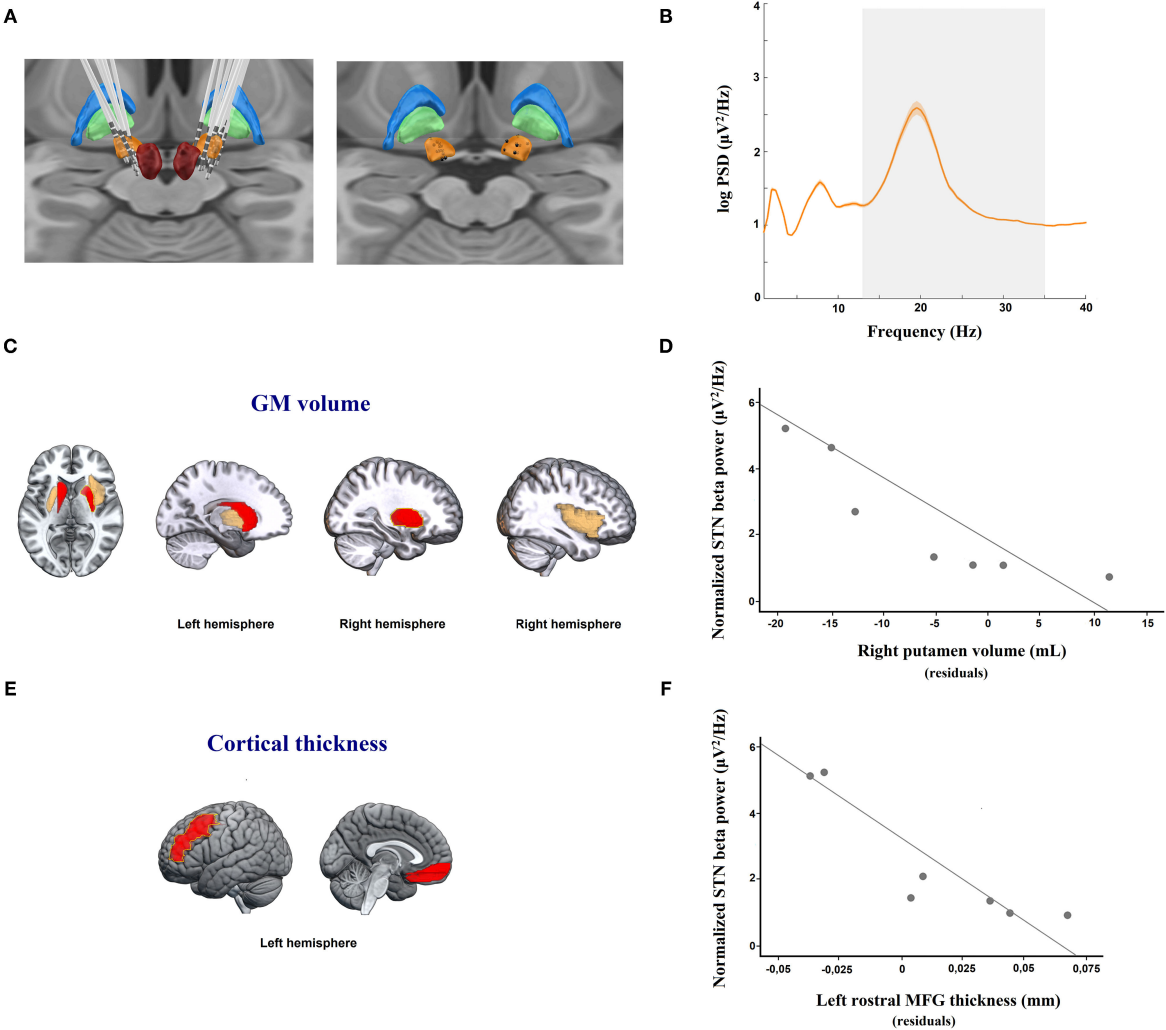
**(A)** DBS electrode location. Images were reconstructed and 3-D visualized using the Lead-DBS toolbox to determine the coordinates of each electrode contact. *Right*: DBS electrode localizations of 7 PD patients; and *left*: simulated location of the position of each bipolar register (black spheres, calculated by averaging the location of each pair of adjacent contacts) in the STN used for LFP analysis. Figures are visualized in the context of a 3D representation of the Morel stereotactic atlas in MNI space (Posterior view). The bilateral subthalamic nucleus (STN; orange), external globus pallidus (GPe; blue), internal globus pallidus (GPi; green), and red nucleus (RN; red) are shown. **(B)** Resting state STN spectral power in PD patients. Averaged (orange solid line) log-transformed power spectral density for STN-LFPs in 7 patients with Parkinson's disease off medication are shown. Orange shaded areas depict S.E.M of the mean for each frequency bin across patients. The x-axis represents frequency (Hz), and the y-axis represents the normalized power spectral density (log PSD, μV^2^/Hz). The gray shaded area indicates the beta frequency spectrum (13–35 Hz). **(C)** Regional analysis of gray matter (GM) volume showing areas negatively correlated, after adjustment for multiple testing, with local field potential (LFP) power in the beta band in the subthalamic nucleus (STN). The yellow line shows the right putamen, which was the only structure retained in the regression model predicting LFP beta activity in the STN (*p* < 0.01). **(E)** Regional analysis of cortical thickness (CT) volume showing areas negatively correlated, after adjustemt for multiple testing, with LFP power in the beta band in the subthalamic nucleus (STN). The yellow line shows the left rostral middle frontal gyrus (MFG), which was the only structure retained in the regression model predicting LFP beta activity in the STN (*p* < 0.01). **(C,E)** The red color denotes brain areas with significant partial correlations after FDR adjustment between regional GM volumes or CT measures and STN LFP beta activity. The yellow color indicates brain areas with a trend toward statistical significance after FDR adjustment between regional GM volumes and STN LFP beta activity. The red and yellows images are overlaid on a standard MNI T1 template. **(D–F)** Scatterplot graphs illustrating the partial correlations between STN LFP beta power and **(D)** right putamen nucleus atrophy and **(F)** left rostral MFG thinning, controlling for age, educational level, and disease duration. For illustration purposes, the y-axis represents the log-transformed power spectral density (log PSD, μV^2^/Hz), and the x-axis of each scatterplot graph represents the averaged residuals of age, educational level, and disease duration of **(D)** regional GM volume in the right putamen and **(F)** CT in the left rostral MFG.

The STN DBS leads were connected by electrode extension cables, which were externalized through the scalp to a Physio16 input box and EGI's Geodesic EEG acquisition system (Electrical Geodesics Inc., EGI®, Eugene, OR). LFP signals were recorded during and digitized with a Net Amps 400 amplifier (EGI®) sampled at 1 kHz using Net Station Acquisition 5.4 software (EGI®). LFPs were obtained by rereferencing adjacent contact pairs of the DBS electrode (bipolar: 0–1, 1–2, and 2–3), which resulted in a maximum of three bipolar channels per electrode (see Figure 1A). Contact 0 is the most ventral, and contact 3 is the most dorsal.

### Electrode Localization and LFP Data Analysis

First, electrodes placed in the STN were individually localized by merging the preoperative MRI and post-operative CT images using Lead-DBS software (36) (www.lead-dbs.org) and following the 2nd pipeline version (37). Thus, all STN leads were visualized post-operatively (see Figure 1A). Consequently, only bipolar channels located inside the STN, according to the DBS electrode reconstructions, were included in the subsequent LFP analysis (see Figure 1A). The LFP data were processed offline using Fieldtrip (38) and custom MATLAB 2018a routines (MathWorks, Natick, MA). The STN LFP data were bandpass-filtered between 0.5 and 98 Hz cutoff frequencies using a fifth-order Butterworth filter. Additionally, a notch filter (band-stop filter at 48–52 Hz, fifth-order Butterworth filters) was applied to remove power line interference at 50 Hz. Subsequently, the continuous recordings were segmented into epochs of 2 s in length. Epochs were rejected when they contained artifacts or non-physiological signals.

Power spectral density (PSD) was calculated by fast Fourier transform (FFT) algorithms using multiple tapers based on discrete prolate spheroidal sequences (DPSS). Beta power frequencies were defined for each participant as the frequency between 13 and 35 Hz at rest. Peak amplitudes for beta power were measured as the highest point of the spectrum in the range of 13–35 Hz. Importantly, no distinction between sensorimotor, associative, or limbic territories of the STN was taken into account for the estimation of LFP power activity, but only contacts including artifact-free data segments that were correctly placed into the STN were considered for further analysis. In 3 patients, bipolar channels of the right or left DBS electrode were specifically excluded from the analysis due to the fact that none of these bipolar channels met the abovementioned LFP inclusion criteria. Further, differences between hemispheres were initially checked by considering only participants in which both hemispheres could be correctly recorded. These analyses confirmed that there were no significant differences regarding the beta frequency peak between right or left hemispheres in our sample patients. As a result, the subsequent analysis distinguishing both hemispheres was not further considered. The resulting data were thus individually averaged across valid contact pairs and hemispheres and were normalized using log transformation for statistical analysis purposes.

### MRI Data Acquisition

Brain MRI data were collected using a 1.5 T scanner (Siemens Magnetom, Erlangen, Germany) ~1–2 weeks before DBS surgery. In addition to MRI sequences necessary for neuronavigation in DBS surgery, 3D T1-weighted MRIs were also acquired at the same time for all participants. This additional structural MRI included (1) a sagittal T1-weighted 3D MPRAGE sequence [repetition time (TR) = 2.200 ms; echo time (TE) = 3.25 ms; flip angle = 8°, matrix = 384 × 512 × 176, and voxel size = 0.5 × 0.5 × 1 mm] and (2) a sagittal FLAIR 3D sequence (TR = 6,000 ms; TE = 358 ms; flip angle = 120°, matrix = 224 × 256 × 160, and voxel size = 1.02 × 1.02 × 1 mm).

### MRI Data Processing to Obtain GM Volume and CT Values

GM volumes were obtained following a voxel-based morphometry (VBM) methodology using the Computation Anatomy Toolbox (CAT-12, version 12.7) *via* the current version of Statistical Parametric Mapping (SPM12; fil.ion.ucl.ac.uk/spm/). For this purpose, a visual inspection from all MRI images was performed initially to ensure that artifacts were absent. Subsequently, images were preprocessed following the standard pipeline recommended in the CAT-12 manual, which includes image bias-field correction, segmentation into GM, white matter (WM) and cerebrospinal fluid (CSF) maps, spatial normalization and modulation of GM maps to the Montreal Neurological Institute (MNI) template.

The obtained tissue compartment volumes were used to calculate total intracranial volume (TIV) as the sum of the GM, WM and CSF volumes. Brain parenchymal fraction (BPF) was calculated as an index of global brain atrophy using the following formula: BPF = GM+WM/TIV (39). GM fraction (GMF) was also calculated as an index of specific global GM atrophy as GMF = GM/TIV (39). Finally, GM regional volumes were extracted using the neuromorphometrics atlas (neuromorphometrics.com/) and were normalized for head size using TIV as a covariate in all subsequent statistical analyses.

CT global and regional values were also obtained with the CAT-12 toolbox following an automated method that relies on a tissue segmentation step to estimate distances between WM and GM voxels using projection-based thickness (40). Regional CT values were obtained using the Desikan-Kiliany atlas (41).

Our study only targeted regions of interest (ROIs) of those cortical-subcortical regions described to be part of the basal ganglia-thalamo-cortical network (42–45), which included ROIs located in the thalamus, basal ganglia, amygdala, hippocampus, cerebellum, cingulate, motor, and prefrontal cortices for analysis of gray matter volumes, and only cingulate, motor, and prefrontal cortices for CT values.

### Statistical Analysis

Statistical analyses were conducted using SPSS v.24 (IBM, Armonk, NY) and custom SPSS syntax routines. Specifically, the non-parametric Spearman partial correlation coefficient (ρ), adjusted for age, educational level, and disease duration, was calculated to separately screen the regional GM volume- and surface-based thickness variables for their relationship with STN LFP beta power. Only those MRI variables (cortical-subcortical atrophy and cortical thickness) that showed a significant correlation after FDR-adjustment (*p* < 0.05) for multiple testing were then entered into two forward linear regression models to determine the strongest MRI predictors of STN LFP beta activity. The residuals obtained from partial correlation, after adjusting for age, educational level, and disease duration, for each significant variable were first saved and then entered as new MRI variables into the regression model as predictors of STN LFP beta (log PSD) power.

## Results

Table 1 summarizes the main clinical characteristics of the PD patient sample. Briefly, PD patients exhibited a mean preoperative UPDRS-III off medication score of 60.1 ± 12.7 and an on medication score of 22.3 ± 10.3. The mean post-operative UPDRS-III off medication score, i.e., immediately prior to the LFP study, was 38.4 ± 13.2. Importantly, our PD sample exhibited preserved cognitive functioning, as revealed by MNSE scores. Additionally, patients showed minimal and mild ranges of depression symptoms based on BDI scores.

### Relationship Between STN LFP Beta Activity and GM Volume and CT

Regarding PSD, the mean STN LFP amplitude spectral density across all subjects showed a single-peaked beta activity of 2.6 μV^2^/Hz in amplitude at 19.53 Hz (see Figure 1B). Specifically, patients presented maximum peaks of beta PSD in the frequency ranges of 15 Hz to 25 Hz with amplitudes between 0.9 and 6.4 μV^2^/Hz. Importantly, 35.7% of the bipolar channels were excluded from the LFP analysis because they were located outside the STN or did not have artifact-free segments.

Spearman partial correlations (adjusted for age and disease duration) revealed that elevated post-operative, but not preoperative, UPDRS-III scores were significantly and positively associated with increases in STN LFP beta power (partial, ρ = 0.831, *p* = 0.041).

To further describe the relationship between regional MRI measures (anatomical regional parcellations for GM atrophy and cortical thickness) and STN LFP beta power, individual tests were assessed using partial correlations adjusted for age, educational level, and disease duration. Exploratory partial correlations results showed that increased LFP beta power in the STN was significantly and negatively correlated to, first, bilateral smaller volume in the putamen, left caudate nucleus, and left external cerebellum, as well as right portions of anterior insula and anterior cingulated gyrus; and second, smaller cortical thickness involving different parcelations of the orbitofrontal gyrus (OFG), left middle frontal gyrus (MFG), and right inferior frontal gyrus (pars triangularis) (see Table 2).

**Table 2:** Spearman partial correlations analysis controlling for age, educational level, and disease duration between log beta power in the STN and both regional gray matter (GM) volume and cortical thickness (CT).

	**Adjusting for age, educational level, and disease duration**		
**Variable**	**Partial Spearman's** **ρ**	***P*** **-values**	**FDR adjusted** ***P*** **<** **0.05**
**Regional GM volume**			
Left caudate nucleus	−0.949	**0.016**	0.048*
Right caudate nucleus	−0.890	0.055^†^	–
Left putamen	−0.923	**0.023**	0.067^†^
Right putamen	−0.977	**0.011**	0.044*
Left external cerebellum	−0.908	**0.045**	–
Right external cerebellum	−0.830	0.085^†^	–
Right anterior insula	−0.948	**0.026**	0.071^†^
Right ACG	−0.934	**0.033**	–
Right frontal operculum	−0.838	0.081^†^	–
**Regional CT**			
Left medial OFG	−0.990	**0.005**	0.037*
Right medial OFG	−0.897	0.052^†^	–
Left rostral MFG	−0.995	**0.001**	0.031*
Left OFG	−0.922	**0.039**	–
Right pars opecularis	−0.868	0.066^†^	–
Right pars triangularis	−0.910	**0.045**	–

*Bold numbers and asterisks indicate significant p-values after FDR adjustment for multiple testing.*

*^†^Indicates statistical trend.*

*ACG, anterior cingulate gyrus; MFG, middle frontal gyrus; OFG, orbitofrontal gyrus*.

Nevertheless, regarding GM atrophy, and after multiple testing adjustment, increased LFP beta power in the STN was only significantly and negatively linked to smaller volume in the right putamen (partial ρ = −0.977; *p* = 0.044) and in the left caudate nucleus (partial ρ = −0.949, *p* = 0.048) (see Table 2; Figure 1C). Only atrophy of the right putamen remained significant in the regression model after adjusting for the effects of age, educational level, and disease duration, and explaining the enhanced LFP beta activity in the STN (*R*^2^ = 0.954, *p* < 0.05) (see Figure 1D).

With respect to cortical thinning, and after multiple testing adjustment, the left rostral MFG (partial ρ = −0.995, *p* = 0.031) and in the left medial OFG (partial ρ = −0.990, *p* = 0.037) were significantly and negatively associated with beta power during resting STN LFP (see Table 2; Figure 1E). Regression analysis, controlling for the effects of age, educational level, and disease duration, confirmed that the cortical thinning of the left rostral MFG accounted for the greatest variance in explaining the STN LFP beta power increase (*R*^2^ = 0.990, *p* < 0.01) (see Figure 1F).

## Discussion

After controlling for age, educational level, and disease duration, the main finding of our study was the association between increases in STN LFP beta oscillatory activity and both reduced GM volumes in dorsal striatum (putamen and caudate nucleus) and reduced CT over the middle prefrontal and orbitofrontal cortices. These results suggest that an ongoing neurodegenerative process would be present at the cortical and subcortical levels that could be linked to a dysfunctional pattern of basal ganglia activity in the beta band in PD patient candidates for STN DBS.

Previous research has broadly demonstrated that the STN LFP beta band may represent a sensitive electrophysiological marker of a patient's clinical symptoms in PD (3, 7, 46), which is correlated with motor symptomatology, such as bradykinesia and rigidity (8, 47, 48), and responds to dopamine replacement therapy and DBS (4, 9, 49–51). Our data showed that STN beta band activity was linked to PD symptom severity as measured by post-operative, but not preoperative, UPDRS-III in the off medication state. This emphasizes that the post-operative UPDRS-III scores, controlled by age and disease duration of the patients and evaluated at the time point of the LFP recordings, could be the most precise way to relate both measurements, given that both are collected under the same experimental conditions and could represent a more precise state of parkinsonian symptomatology linked to the impact of the stun effect, which may influence oscillatory activity patterns in the STN (52). Nevertheless, despite this insertional effect, the patient clinical improvement commonly observed and induced by the electrode insertion differs substantially from that obtained with dopaminergic treatment (6) and ON DBS (7).

The current study also demonstrated that volume reduction in the dorsal striatum accounted for somewhat more variance than cortical volume in explaining beta band STN LFP activity. Regarding GM volume, consistent findings have pointed to progressive atrophy accumulation in subcortical areas, such as the putamen and caudate, and in both the early and middle stages of PD (53, 54). Importantly, the putamen, which is a central projection site of the cortical inputs into the basal ganglia, and its activity is primarily movement-related, has been frequently considered one of the first structure that shows both volume and shape variations in PD (44, 45, 55–57), so that putaminal alterations can decidedly contribute to the pathophysiology of cortico-basal ganglia motor loops. Moreover, caudate volume loss has aroused great interest as an MRI biomarker of disease progression from early PD stages (24, 28, 58) and is also linked to conversion to mild cognitive impairment or dementia (27, 28, 59). Therefore, the putamen and caudate (and cerebellum) are considered primarily related to motor function, such as coordination, planning, execution, and movement regulation, and are also increasingly recognized as relevant hubs related to inhibitory control of action, learning, and cognition (60). Our results are consistent with this evidence and underscore that decreased dopaminergic inputs to the basal ganglia, particularly to the dorsal striatum, and likely due to progressive accumulation of atrophy as a consequence of the evolution of the disease, promote synchronized beta LFP activity in the STN and abnormal pallido-striatal feedback (16–18, 61, 62) and could lead to characteristic parkinsonism symptoms (7, 63).

Despite its relevance as a potential neurodegenerative biomarker of disease progression, little emphasis has been previously given to CT and its likely relationship with beta oscillatory activity in the STN. After controlling for the age, educational level, and disease duration, the current study revealed that CT reductions may be associated with enhanced STN beta activity, mainly involving regions in the middle prefrontal and orbitofrontal cortices. In fact, cortical thinning involving prefrontal regions has been previously reported in PD patients. Furthermore, there is converging evidence stressing the so-called “limbic hyperdirect pathway” between the ventral and medial OFG and the STN (64). Hence, enhanced STN beta rhythm may also be linked to a pattern of regional cortical thinning that would correspond to specific nodes of large-scale cortical networks structurally connected to basal ganglia and functionally specialized in limbic processing (e.g., medial PFC/OFC), including language-related regions (pars triangularis and pars opecularis).

It is worth mentioning some limitations to this observational pilot study. First, despite the robustness of our results, the main limitation was the small patient sample size. Increasing the sample size in future studies would allow us to better address the current findings, enabling us to assess different clinical subgroups according to PD symptoms, which would enhance the significance of the present results. Second, our findings are correlational, so we cannot establish causation. Therefore, it is recommended to focus on establishing causal connections to related STN beta activity to neurodegenerative states of the specific brain areas. Third, we did not investigate the lower and upper ranges of the STN beta frequency band or from different territories of the STN, which may have somewhat different functional significance. For example, it has been frequently demonstrated that low beta activity is suppressed by current conventional dopaminergic or DBS therapies, allowing alleviation of PD symptoms, which would mostly reflect a pathological STN-striatal feedback mechanism, while high beta activity is relatively less modulated after dopaminergic medication withdrawal and seems to be more strongly coupled with cortical activity (8, 47, 65–67). Further studies should better address the association between regional cortical-subcortical volume-surface-based MRI measures and the subranges of the STN beta band from three functional territories of the human STN (sensorimotor, associative, limbic). This would provide more precise insight into the functional mapping of STN activity and its relationship with neurodegenerative processes of cortical-subcortical large-scale networks in PD.

## Conclusions

The results of this pilot study support the investigation of how exaggerated synchrony at beta frequencies within the STN might be accompanied by both GM volume reductions in dorsal striatal motor circuits and more widespread thinning of prefrontal associative territories, and suggest that this association remains to be elucidated in detail. Taken together, these findings support the hypothesis that spreading neurodegeneration of nigrostriato regions may also functionally and structurally impair distinct striato-cortical connections, likely contributing to the malfunctioning of oscillatory activity of the STN observed in PD. The use of multiple brain markers resulting from multimodal imaging techniques may provide new insights into the neuroanatomical and pathophysiological mechanisms associated with the underlying disease.

## Data Availability Statement

The original contributions presented in the study are included in the article/supplementary material, further inquiries can be directed to the corresponding author.

## Ethics Statement

All the participants provided written informed consent for the STN DBS and LFP study before participation in accordance with the Declaration of Helsinki. The study was approved by the Andalusian Biomedical Research Ethics Committee (Ref.: PI-0025-2017/Acta03/2018).

## Author Contributions

JJG-R conceived and designed the study and drafted the manuscript. FS, ÁJC-G, and JJG-R wrote the manuscript, performed statistical analysis, and interpreted results of study. ÁJC-G and AZ collected MRI data. RR-L and RE-R recruited and performed clinical evaluations of patients, conducted patient testing during and after the surgery, and contributed to interpretation of the results. JR-D performed the DBS surgeries and contributed to data collection. EL-S and ÁJC-G provide clinical and neuropsychological information. FS and FL-S prepared the recording setting and collected the LFP data. FS performed the LFP analysis and electrode localization. ÁJC-G performed MRI analysis. FS, ÁJC-G, RR-L, EL-S, FL-S, and JJG-R edited and revised the manuscript. All authors approved the final version of the manuscript.

## Funding

This work has been supported by the Spanish Ministry of Science, Innovation and Universities (MICINN) under Grants (RYC-2015-18467; PSI2017-85951-R) and by the European Regional Development Fund through the Andalusian Ministry of Health and Families under Grants (PI-0025-2017; PI-0034-2019).

## Conflict of Interest

RR-L and RE-R received speaker fees and travel support from Teva, AbbVie, Zambon, BIAL, Italfarmaco, Biogen, Merck Serono, Novartis, Sanofi, and Roche. JR-D received travel support and training from Medtronic, but not related to this study. The remaining authors declare that the research was conducted in the absence of any commercial or financial relationships that could be construed as a potential conflict of interest.

## Publisher's Note

All claims expressed in this article are solely those of the authors and do not necessarily represent those of their affiliated organizations, or those of the publisher, the editors and the reviewers. Any product that may be evaluated in this article, or claim that may be made by its manufacturer, is not guaranteed or endorsed by the publisher.
